# Comparable outcomes following combined ACL and ALL reconstruction using a 1‐strand versus 2‐strand back‐and‐forth technique: Propensity score matched study

**DOI:** 10.1002/jeo2.70658

**Published:** 2026-03-07

**Authors:** Guillaume Andre, Damien Block, Olivier Gosselin, Jean Hennequin, ELSAN Working Group, Floris van van Rooij, Chinyelum Agu, Julien Uhring

**Affiliations:** ^1^ COUBORTHO, Centre Coubertin/Hôpital‐clinique Claude Bernard, ELSAN Metz France; ^2^ ReSurg SA Nyon Switzerland

**Keywords:** ACL, ALL, clinical outcomes, dyneelax, knee laxity, reconstruction techniques

## Abstract

**Purpose:**

To compare post‐operative laxity and clinical outcomes following combined anterior cruciate ligament (ACL) and anterolateral ligament (ALL) reconstruction with hamstring grafts using a 1‐strand versus 2‐strand back‐and‐forth technique.

**Methods:**

The authors retrospectively assessed a consecutive series of 348 patients (348 knees), that underwent combined ACL and ALL reconstruction using the 2‐strand technique, and 130 patients (130 knees), that underwent combined ACL and ALL reconstruction using 1‐strand technique between January 2020 and December 2022. Propensity score matching was performed to establish two comparable study cohorts. Patients underwent pre‐ and postoperative assessment by one observer using Knee injury and Osteoarthritis Outcome Score (KOOS‐12), Lysholm, Tegner, and ACL‐RSI scores. Physical examinations included a jerk test, and the attribution of a Lachman grade. Postoperatively, patients were assessed using the Dyneelax arthrometer on both knees to measure the differential laxity in anterior translation (AT) and internal rotation (IR).

**Results:**

Propensity score matching resulted in 70 patients in the 2‐strand group, and 70 patients in 1‐strand group. Post‐operatively, 18 patients were lost to follow up, and five patients underwent revision surgery due to a retear. This left a final cohort of 58 patients in the 2‐strand group, aged 30.5 ± 8.1 (range, 18–52), with a BMI of 25.8 ± 4.3 (range, 20–37.8), and 59 patients in the 1‐strand group, aged 30.7 ± 10.7 (range, 15–55), with a BMI of 26.5 ± 3.9 (range 16.9–38.6). At a mean follow‐up of 2.4 ± 0.4 years, there were no significant differences between the groups in terms of differential laxity in AT, IR, KOOS‐12, ACL‐RSI, Lysholm, Tegner scores, and retear rates.

**Conclusion:**

There was no significant difference between the 1‐ and 2‐strand techniques for combined ACL and ALL reconstruction in terms of post‐operative laxity, functional and clinical outcomes, including return to sport and Tegner activity level, suggesting that both techniques are equally effective in restoring knee stability and function.

**Level of Evidence:**

Level IV, case series.

## Graphical abstract

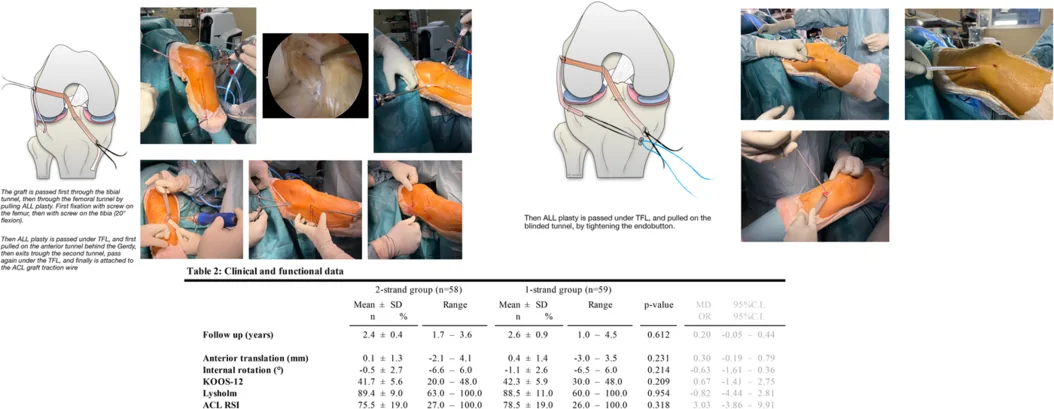

**Abbreviations**
ACL: anterior cruciate ligament
ACLR: anterior cruciate ligament reconstruction
ALL: anterolateral ligament
AMI: arthrogenic muscular inhibition
AT: anterior translation
BMI: body mass index
GJL: Generalised Joint Laxity
GT: gracilis tendon
IR: internal rotation
KOOS: Knee injury and Osteoarthritis Outcome Score
ST: semitendinosus

## INTRODUCTION

Injuries of the anterior cruciate ligament (ACL) are some of the most common knee injuries, especially in an active population. Isolated ACL reconstruction (ACLR) is the gold standard treatment, with satisfactory clinical scores and low retear rates [8, 25], restoring stability and function [29]. However, more active patients may obtain limited recovery due to residual laxity and rotational instability, often leading to re‐injury or retears, despite successful surgeries [15, 29, 31, 34]. Various techniques and graft options have been investigated to mitigate these functional impairments [2, 6, 13, 19, 21, 24, 33], such as reconstruction of the anterolateral ligament (ALL) [7, 9, 11, 18, 25], involving the semitendinosus tendon (ST), and gracilis tendon (GT) which can be left inserted on the tibia or detached [10, 26, 27, 31].

Combined reconstruction of the ACL and ALL has demonstrated promising outcomes in active patients [16], however, to date, there is limited evidence on the optimal lateral procedure to reduce post‐operative residual anterior and rotational laxity. Reconstruction of the ALL has demonstrated many benefits, such as reduction of physiological laxity in skeletally immature patients [4, 12], better ACLR graft incorporation [5], and an improvement in rotational stability [19] as well as ACLR graft survival [17, 28]. The senior surgeon has been performing ACLR with ALL reconstruction according to the technique published by Sonnery‐Cottet et al. [31], using a pedicled ST and GT graft for a combined ACL and ALL reconstruction using a 2‐strand technique back‐and‐forth technique through two tibial tunnels. Recently, the senior surgeon has adapted another combined ACL and ALL reconstruction technique published by Coulet et al. [10], by detaching the ST and GT tibial insertions, using a single tunnel for the ALL reconstruction, which could reduce chances of misdrilling, and provides a more aesthetic outcome, as the grafts are palpable through the skin. Investigating laxity, and anterior translation can shed light on the outcomes of the two techniques. Furthermore, clinical outcomes and return to sport may also aid clinicians in surgical decision‐making to limit ACL retears, and reassure patients on post‐operative expectations following ACL reconstruction.

The purpose of the present study is therefore to compare post‐operative laxity following combined ACL and ALL reconstruction with hamstring grafts using a using a 1‐strand versus 2‐strand back‐and‐forth technique. The hypothesis is that both techniques will result in similar outcomes.

## METHODS

The authors retrospectively compared a consecutive series of 348 patients (348 knees) that underwent combined ACL and ALL reconstruction using the 2‐strand technique, and 130 patients (130 knees), that underwent combined ACL and ALL reconstruction using 1‐strand technique between January 2020 and December 2022. All patients that had a primary ACL tear, underwent ACL reconstruction with an ALL reconstruction, with no specific indications. The inclusion criteria were, patients that underwent primary ACLR with ALL reconstruction. Exclusion criteria were, patients with surgical antecedents on the ipsilateral knee, contralateral ACL tear and patients who required additional osteotomy, chondral reconstruction, or treatment of the collateral knee ligaments, were excluded. All patients provided informed consent, and IRB was not requested as the assessments were part of the routine care.

### Pre‐operative assessment

Prior to surgery, assessments were performed by one observer, where patients were required to complete the Knee injury and Osteoarthritis Outcome Score (KOOS‐12) [23], the Lysholm score [3], and the Tegner activity level [3] (these questionnaires were completed in French, but the French version of the Lysholm score and Tegner activity level are yet to be validated). Furthermore, clinical examinations were performed to evaluate mobility, pain, and arthrogenic muscular inhibition (AMI) [30]. The assessment was finalised with the execution of a pivot‐shift jerk test, and the attribution of a Lachman grade [14].

### Surgical technique

The ACL reconstruction combined with ALL reconstruction using a 2‐strand technique through two tibial tunnels, and has been previously published by Sonnery‐Cottet et al. [31], and consists of the following steps: The ST and GT grafts are harvested, and the tibial tunnel for the ACLR is drilled. The ALL graft attachment is prepared using a proximal drill hole situated in the site of the Segond fracture and a distal hole located anterior and inferior to this site, both to ensure a good osseous tunnel and to replicate the anatomy and isometry of the ALL. The femoral tunnel for the ACLR is drilled. Then the grafts for the ACL (3ST + 1GT) and ALL (GT) are prepared, and the ACL and ALL reconstructions are performed using a single loop. No cartilage procedures were performed.

The ACL reconstruction combined with ALL reconstruction using one strand through one tibial tunnel, has been adapted from Coulet et al. [10], and is consists of the following steps: The ST and GT grafts are harvested with detachment of the tibial insertions, and the tibial tunnel for the ACLR is drilled. The ALL graft attachment is prepared using a single medial drill hole equidistant between the Gerdy tubercle and the head of the fibula, approximately 2 cm above the fibular head and exiting 2 cm below the ACLR tibial tunnel laterally. The femoral tunnel for the ACLR is drilled. Then the grafts for the ACL (3ST + 1GT) and ALL (GT) are prepared, and the ACL and ALL reconstructions are performed. No cartilage procedures were performed.

### Postoperative rehabilitation

All patients the same standardised postoperative protocol, that did not limit postoperative weight‐bearing, and walking was allowed immediately. Patients used a knee brace. Physiotherapy started after 1 week. Cycling and swimming were allowed after 6 weeks. Running and fitness was allowed after 3–4 months. Isokinetic evaluation was performed after 6 months, and if it was deemed sufficient, patients were allowed to resume football and return to impact and rotational sports.

### Post‐operative assessment

Post‐operatively, KOOS‐12, Lysholm, Tegner, and ACL‐RSI scores were collected by 1 observer. Physical examinations performed by the clinician included a pivot‐shift test, and the attribution of a Lachman grade (subjective). Patients were then assessed using the Dyneelax ® arthrometer (Genourob, France) as an objective assessment on both knees to measure the differential laxity (difference between the ipsilateral and contralateral knees) in AT and IR.

### Statistical analysis

**Figure 1:**
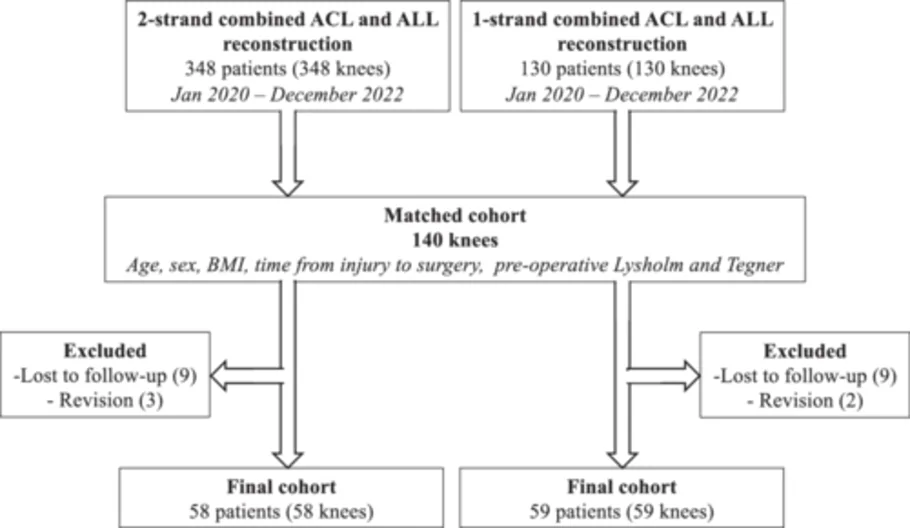
Flowchart of the study cohort. ACL, anterior cruciate ligament; ALL, anterolateral ligament.

Propensity score matching was performed to define two comparable study cohorts and to perform postoperative assessment of matched patients (Figure 1). Propensity scores were estimated for each patient using a logistic regression model considering age, sex, BMI, time from injury to surgery, pre‐operative Lysholm and Tegner. A 1:1 nearest‐neighbour algorithm with a caliper of 0.01 was applied to match patients using their corresponding propensity scores. A priori sample size calculation was performed, and assuming a postoperative Lysholm score of 89 points, a difference of 5.5 points (similar to the MCID [20]), would require 47 patients per group, with a power of 90%. Descriptive statistics were used to summarise the findings, and Shapiro–Wilk tests were used to assess the normality of data distributions. For Gaussian distributed continuous data, differences between groups were evaluated using unpaired t‐tests. For non‐Gaussian continuous data, differences between groups were evaluated using Wilcoxon rank sum tests (Mann–Whitney *U* test). For categorical variables, comparisons between groups were performed using the Chi‐square test or ANOVA test Statistical analyses were performed using R version 4.2.3 (R Foundation for Statistical Computing, Vienna, Austria).

## RESULTS

Propensity score matching resulted in 70 patients in the 2‐strand group, and 70 patients in 1‐strand group. Post‐operatively, 18 patients were lost to follow up, and five patients underwent revision surgery due to a retear (three in the 2‐strand group, and two in the 1‐strand group; Figure 1). This left a final cohort of 58 patients in the 2‐tunnel group, aged 30.5 ± 8.1 (range, 18–52), with a BMI of 25.8 ± 4.3 (range, 20–37.8), and 59 patients in the 1‐strand group, aged 30.7 ± 10.7 (range, 15–55), with a BMI of 26.5 ± 3.9 (range 16.9–38.6) (Table 1). In the 2‐strand group, 24 patients required meniscal sutures, while in the 1‐strand group 29 patients required meniscal sutures.

**Table 1:** Patient characteristics.

	Initial cohort (*n* = 140)					Final cohort (*n* = 117)				
	2‐strand group (*n* = 70)		1‐strand group (*n* = 70)			2‐strand group (n = 58)		1‐strand group (*n* = 59)		
	Mean ± SD		Mean ± SD			Mean ± SD		Mean ± SD		
	*n* (%)	Range	*n* (%)	Range	*p*‐value	*n* (%)	Range	*n* (%)	Range	*p*‐value
Female	17 (24%)		15 (21%)		*0.841*	13 (22%)		12 (20%)		*0.825*
Height	1.8 ± 0.1	1.5–1.9	1.8 ± 0.1	1.6–2.0	*0.615*	1.8 ± 0.1	1.5–1.9	1.8 ± 0.1	1.6–2.0	*0.486*
Weight	79.6 ± 15.3	50.0–128.0	82.4 ± 13.8	56.0–125.0	*0.179*	79.3 ± 15.0	50.0–128.0	82.5 ± 14.3	56.0–125.0	*0.181*
BMI	25.9 ± 4.3	20.0–37.8	26.5 ± 3.8	16.9–38.6	*0.123*	25.8 ± 4.3	20.0–37.8	26.5 ± 3.9	16.9–38.6	*0.159*
Age at trauma	29.3 ± 8.2	16–52	29.0 ± 10.4	15–55	*0.524*	29.7 ± 8.1	18–52	30.0 ± 10.7	15–55	*0.777*
Age at surgery	30.2 ± 8.1	16–52	29.8 ± 10.4	15–55	*0.505*	30.5 ± 8.1	18–52	30.7 ± 10.7	15–55	*0.764*
Time from injury to surgery (months)	10.8 ± 20.3	1.2–112.2	9.8 ± 15.0	0.7–79.6	*0.163*	9.4 ± 18.0	1.2–112.2	8.8 ± 14.7	0.7–79.6	*0.135*
Right side	28 (40%)		29 (41%)		*1.000*	26 (45%)		26 (44%)		*1.000*
Dominant side	31 (44%)		34 (49%)		*0.735*	28 (48%)		30 (51%)		*0.854*
Previous meniscus surgery	1 (1%)		0 (0%)		*1.000*	1 (2%)		0 (0%)		*1.000*
Work accident	4 (6%)		2 (3%)			4 (7%)		2 (3%)		*0.439*
Profession					*0.372*					*0.565*
Unemployed	4 (6%)		1 (1%)			4 (7%)		0 (0%)		
Sedentary	21 (30%)		33 (47%)			17 (29%)		28 (47%)		
Manual	41 (59%)		31 (44%)			34 (59%)		28 (47%)		
Repetitive	4 (6%)		5 (7%)			3 (5%)		3 (5%)		
Type of trauma					*0.636*					*0.543*
Fall	10 (14%)		8 (11%)			8 (14%)		6 (10%)		
Sport	58 (83%)		59 (84%)			48 (83%)		51 (86%)		
No trauma/does not know	2 (3%)		3 (4%)			2 (3%)		2 (3%)		

Abbreviation: SD, standard deviation.

### Laxity assessment

At a mean follow‐up of 2.4 ± 0.4 years, the 2‐strand group had a mean differential laxity in AT of 0.1 ± 1.3 mm, and IR of −0.5° ± 2.6 (Table 2). In comparison, at a mean follow‐up of 2.6 ± 0.9 years, patients in the 1‐strand group had a mean differential laxity in AT of 0.4 ± 1.4 mm, and IR of −1.1° ± 2.6. No significant differences were found between the two groups postoperative anterior laxity (Lachman grade (*p* = 0.302) and Dyneelax test), IR laxity (pivot‐shift grade (*p* = 0.271) and Dyneelax test (*p* = 0.214)) and retear rate.

**Table 2:** Clinical and functional data.

	2‐strand group (*n* = 58)		1‐strand group (*n* = 59)				
	Mean ± SD		Mean ± SD			MD	
	*n* (%)	Range	*n* (%)	Range	*p*‐value	OR	95% CI
Follow‐up (years)	2.4 ± 0.4	1.7–3.6	2.6 ± 0.9	1.0–4.5	0.612	0.20	−0.05 to 0.44
Pre‐operative							
Instability	46 (79%)		47 (80%)		1.000		
Pain	22 (38%)		22 (37%)		1.000		
Flexum (extension limitation)	2 (3%)		5 (8%)		0.439		
Hyperlaxity (hyperextension)	16 (28%)		15 (25%)		0.836		
KOOS‐12	24.9 ± 7.6	10.0–43.0	24.8 ± 8.7	7.0–45.0	0.857	−0.12	−3.07 to 2.83
Lysholm	51.4 ± 16.9	13.0–84.0	53.4 ± 23.6	4.0–94.0	0.588	2.06	−5.35 to 9.48
Tegner					0.858		
0	1 (2%)		0 (0%)				
3	2 (3%)		2 (3%)				
4	13 (22%)		10 (17%)				
5	7 (12%)		5 (8%)				
6	0 (0%)		1 (2%)				
7	6 (10%)		8 (14%)				
8	2 (3%)		2 (3%)				
9	27 (47%)		31 (53%)				
Lachman Grade					0.656		
1	10 (17%)		8 (14%)				
2	37 (64%)		36 (61%)				
3	11 (19%)		15 (25%)				
Pivot‐shift (Jerk test)					0.259		
0	21 (36%)		19 (32%)				
1	8 (14%)		17 (29%)				
2	24 (41%)		19 (32%)				
3	5 (9%)		4 (7%)				
Post‐operative							
Anterior translation (mm)	0.1 ± 1.3	−2.1 to 4.1	0.4 ± 1.4	−3.0 to 3.5	0.231	0.30	−0.19 to 0.79
Internal rotation (°)	−0.5 ± 2.7	−6.6 to 6.0	−1.1 ± 2.6	−6.5 to 6.0	0.214	−0.63	−1.61 to 0.36
KOOS‐12	41.7 ± 5.6	20.0–48.0	42.3 ± 5.9	30.0–48.0	0.209	0.67	−1.41 to 2.75
Lysholm	89.4 ± 9.0	63.0–100.0	88.5 ± 11.0	60.0–100.0	0.954	−0.82	−4.44 to 2.81
ACL RSI	75.5 ± 19.0	27.0–100.0	78.5 ± 19.0	26.0–100.0	0.318	3.03	−3.86 to 9.91
Tegner					0.246		
3	1 (2%)		6 (10%)				
4	12 (21%)		9 (15%)				
5	8 (14%)		3 (5%)				
6	6 (10%)		7 (12%)				
7	11 (19%)		17 (29%)				
8	2 (3%)		2 (3%)				
9	18 (31%)		15 (25%)				
Lachman Grade					0.302		
0	50 (86%)		43 (73%)				
1	7 (12%)		13 (22%)				
2	1 (2%)		2 (3%)				
Pivot‐shift (Jerk test)					0.271		
0	44 (76%)		48 (81%)				
1	10 (17%)		10 (17%)				
2	3 (5%)		0 (0%)				
3	1 (2%)		0 (0%)				

Abbreviations: CI, confidence interval; KOOS, Knee injury and Osteoarthritis Outcome Score; OR, odds ratio; SD, standard deviation.

### Patient reported outcomes

Patients in the 2‐strand group reported a mean KOOS‐12 of 41.7 ± 5.6, Lysholm score of 89.4 ± 9.0, and ACL‐RSI score of 75.5 ± 19.0. Tegner scores ranged from 3 to 9, with 53% of patients reaching a score ≥7. In comparison, patients in the 1‐strand group reported a mean KOOS‐12 of 42.3 ± 5.9, Lysholm score of 88.5 ± 11.0, and ACL‐RSI score of 78.5 ± 19.0. Furthermore, Tegner scores ranged from 3 to 9, with 58% of patients reaching a score ≥7. There were no significant differences between the two groups in terms of KOOS‐12 (*p* = 0.209), Lysholm (*p* = 0.954), ACL‐RSI (*p* = 0.318) or Tegner scores (*p* = 0.246).

## DISCUSSION

The main findings of the present study are that there is no significant difference in postoperative laxity between the 1‐strand and 2‐strand techniques for combined ACL and ALL reconstruction. In addition to comparable laxity, functional and clinical outcomes, including return to sport and Tegner activity level were also similar between groups. These findings suggest that both techniques are equally effective in restoring knee stability and function. Therefore, the results support the initial hypothesis that both techniques would yield similar outcomes, despite the technical differences.

Reconstruction of the ALL while performing ACLR has gained popularity, particularly for active patients, as single bundle ACLR has shown to lead to limited recovery due to residual anterior and rotational laxity [15, 29, 31, 34]. With that in mind, several techniques for lateral reinforcement of the knee have been investigated [2, 6, 7, 9, 11, 13, 18, 19, 21, 24, 25, 33]. The present study compared two similar techniques: the 1‐strand versus 2‐strand technique. The one strand through one tunnel technique is easier to perform, as it only requires drilling of a single tibial tunnel, thereby reducing chances of misplacement. In contrast, the 2‐strand through two tunnel technique may result in poor placement of the anterior tunnel if drilled too close to the Gerdy tubercule. Additionally, creating two converging tunnels could compromise bone integrity, and result in bone collapse between the two tunnels [10, 31]. Furthermore, as the ALL reconstruction is palpable through the skin, using one strand leads to more aesthetic outcomes, than two strands. Despite these technical differences, no clinical differences were observed between the two groups in terms of post‐operative laxity, functional and clinical scores, including return to sport, and Tegner activity level. The comparable outcomes may be related to the portion of graft fixed immediately behind Gerdy's tubercule, which likely plays a key role in extra‐articular stabilisation. In the 2‐strand technique, the posterior graft section is engaged later due to its orientation, which may delay the onset of tension, but ultimately results in similar stabilisation. These findings were supported by functional assessments such as the pivot shift, and the Dyneelax ® measurements, which demonstrated no difference in the postoperative rotational stability between the two techniques.

In addition to the number and position of the tunnels used for the two techniques assessed in the present study, the procedures also differed with regards to the preservation or detachment of the tibial insertions of the ST and GT. Indeed, the 1‐strand technique detaches both the ST and GT when preparing the ACL and ALL graft, while in the 2‐strand technique, both tendons are left pedicled. The literature suggests that preserving the tibial insertion could result in better maturation of the intra‐articular reconstruction, and could provide additional natural reinforcements counteracting sheer stress on tibial interference screws, potentially improving graft stability [1, 5, 22, 32]. Vari et al. [32] found that pedicled grafts resulted in better remodelling than non‐pedicled grafts in standard ACLR. The present study, however, showed no significant difference in the post‐operative Lachman test and AT measurements between the two techniques, suggesting that in mid‐term follow‐up, clinical benefits of preserving the tibial insertions may be limited. A possible explanation could be that performing additional ALL reconstruction, regardless of the tibial insertions, would reduce stresses during rotational movements, therefore allowing better maturation of the procedure. Cavaignac et al. [5] found that ACLR procedures with additional lateral extra‐articular tenodesis (LET) resulted in better maturation and incorporation on MRI than ACLR procedures alone. Future studies assessing mid to long‐term outcomes of the two techniques on imagery could clarify whether pedicled grafts result in better structural integrity than detached grafts when performing an additional ALL reconstruction.

The present study assessed range of motion using the Dyneelax ® arthrometer on both knees to measure the differential laxity between the ipsilateral and contralateral knees in AT and IR. An important observation was that the mean post‐operative IR was decreased compared to the natural IR measured on the contralateral knee. While reduction in IR could be beneficial for graft survival in short to mid‐term follow‐up, it could result in an increased stress on the lateral compartment on longer term follow‐up, leading to cartilage wear and degeneration. Patients with generalised joint laxity (GJL) may be more vulnerable to excessive stress on the lateral compartment. Future studies should assess the relationship between IR restriction and long‐term development of cartilage degeneration in the general population, and in patients with GJL.

The findings of the present study should be interpreted with the following limitations in mind. First, 1:1 patient matching resulted in a notable reduction of the cohort size due to the important difference in time from injury to surgery between the two groups in the unmatched cohort, and was not adjusted for preoperative characteristics such as, meniscal injury, laxity, or preoperative pivot‐shift, making residual confounding likely. Second, the present study investigates short‐ to mid‐term outcomes, and would benefit from a longer follow‐up to assess comparability of the two techniques over time, and effects of reduced IR in the general population and in patients with GJL. Third, the tunnel placement was not analysed. Inappropriate tunnel placement could have some influence on excess stress on the lateral compartment. Including this variable in future analyses could allow detection of a potential association with long‐term cartilage deterioration, and therefore, elimination of a confounding factor in long‐term outcomes. Fourth, preoperative laxity assessment was only performed by one observer, which could have introduced a substantial risk of measurement bias. Furthermore, there was no objective preoperative laxity assessment, making it difficult to compare the degree of improvement between the techniques. Fifth, the authors did not note detailed information about concomitant pathologies, such as tear types, repair techniques, or cartilage status. Finally, radiographic evaluation of the tibial slope was not assessed, and osteoarthritis revealed no progression of osteoarthritis, but this was only assessed at 6 months following surgery, and could lead to different results in the long‐term.

## CONCLUSION

There was no significant difference between the 1‐strand and 2‐strand techniques for combined ACL and ALL reconstruction in terms of post‐operative laxity, functional and clinical outcomes, including return to sport and Tegner activity level, suggesting that both techniques are equally effective in restoring knee stability and function.

## AUTHOR CONTRIBUTIONS

**Guillaume Andre**: Procurement of funding, study design, data collection, interpretation of findings and manuscript writing. **Damien Block**: Manuscript validation. **Olivier Gosselin**: Manuscript validation. **Jean Hennequin**: Manuscript validation. **Julien Uhring**: Manuscript validation. **Floris van Rooij** and **Chinyelum Agu:** Methodology, data curation, formal analysis, statistical analysis, manuscript writing.

## ELSAN WORKING GROUP

Floris van Rooij and Chinyelum Agu, ReSurg SA, Nyon, Switzerland.

## CONFLICT OF INTEREST STATEMENT

Guillaume Andre report consulting fees from Vims/Zimmer and United. Julien Uhring report consulting fees from Conmed and Moveup. The other authors declare no conflicts of interest.

## ETHICS STATEMENT

All patients provided informed consent.

## Supporting information


**Supplementary figures 1:** 1‐Strand surgical technique.

**Supplementary figures 2:** 2‐Strand surgical technique.
