# Supplementary figures and images for: Comparable outcomes following combined ACL and ALL reconstruction using a 1‐strand versus 2‐strand back‐and‐forth technique: Propensity score matched study
Source: J Exp Orthop. 2026 Mar 7;13(1):e70658. doi: 10.1002/jeo2.70658 (PMC12966925; doi:10.1002/jeo2.70658)

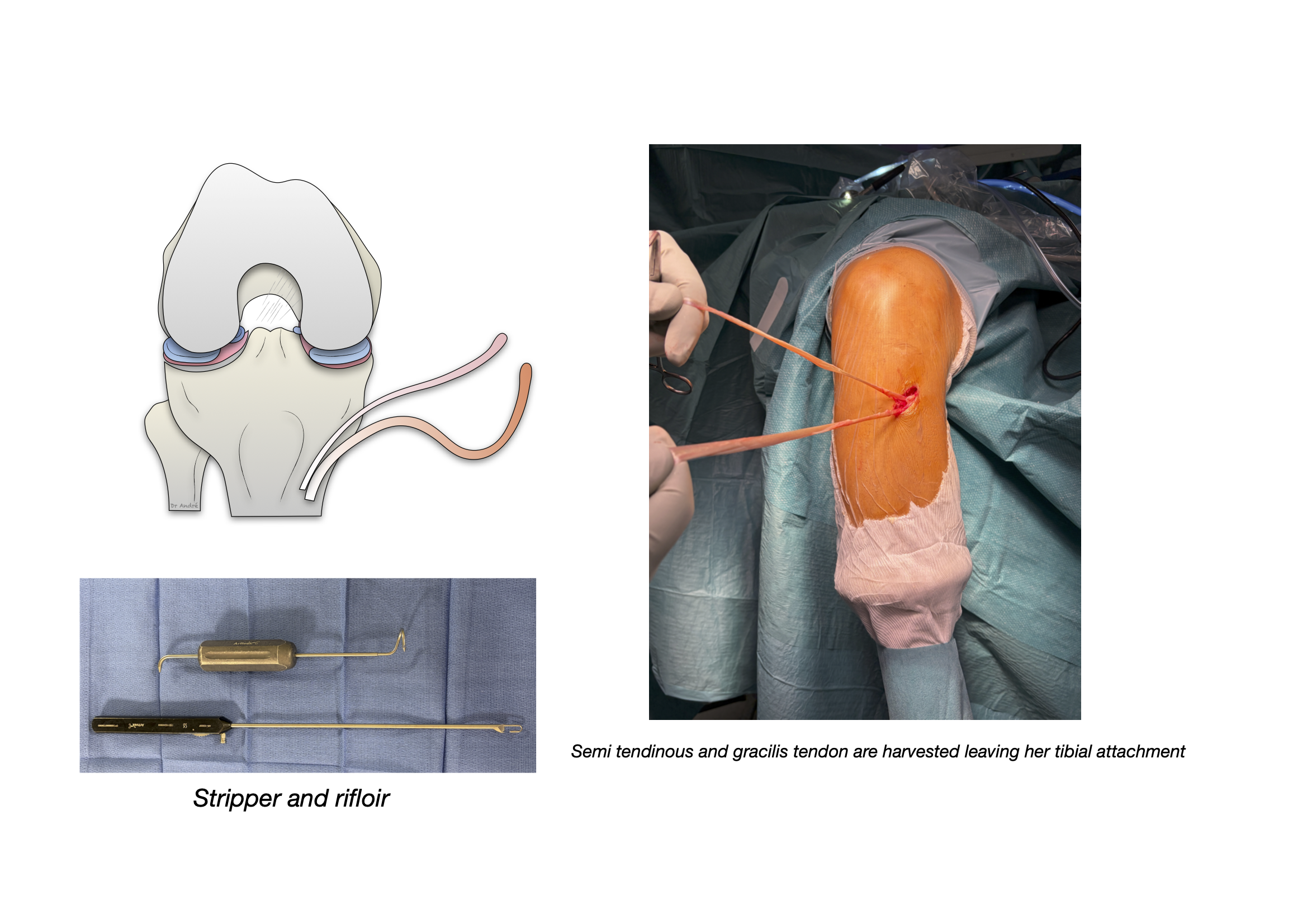

Supplement: Supplementary file 1 — Supplementary figures 1: 1‐Strand surgical technique. [file JEO2-13-e70658-s001.png]

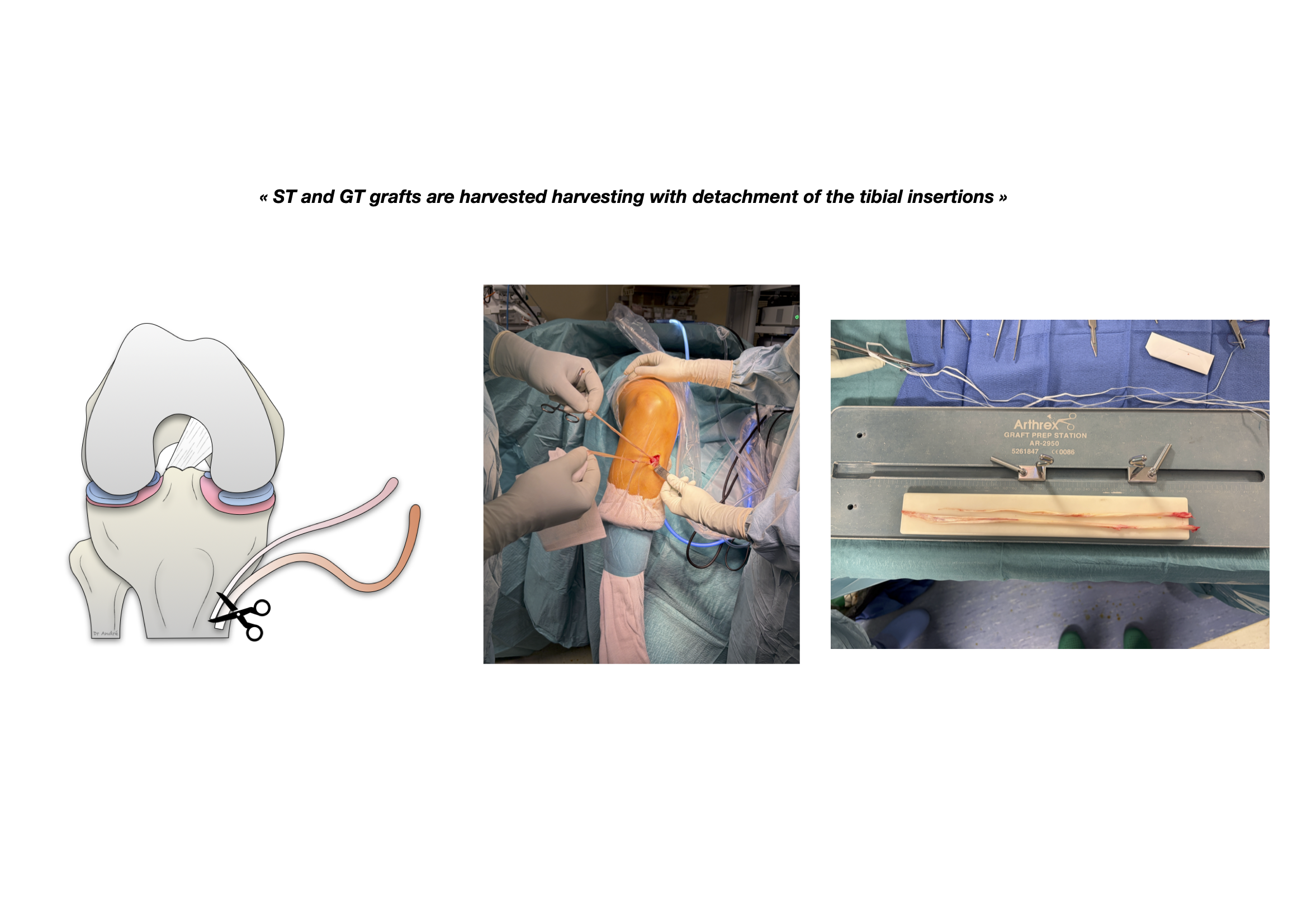

Supplement: Supplementary file 2 — Supplementary figures 2: 2‐Strand surgical technique. [file JEO2-13-e70658-s002.png]
